# Use and safety of peripherally inserted central catheters and midline catheters in palliative care cancer patients: a retrospective review

**DOI:** 10.1007/s00520-023-08045-2

**Published:** 2023-09-19

**Authors:** Eva Gravdahl, Siri Steine, Knut Magne Augestad, Olav Magnus Fredheim

**Affiliations:** 1 Department of Palliative Medicine, Akershus University Hospital, Sykehusveien 25, 1478 Lørenskog, Norway; 2 Institute of Clinical Medicine, University of Oslo, Problemveien 7, 0315 Oslo, Norway; 3 Department of Gastrointestinal Surgery, Akershus University Hospital, Oslo, Norway; 4 Department of Surgery, Helgelandssykehuset, Prestmarkveien 1, 8800 Sandnessjøen, Norway

**Keywords:** Cancer, Drug administration, Palliative care, Intravenous access, Peripherally inserted central catheter, Midline catheter

## Abstract

**Purpose:**

Some cancer patients in palliative care require intravenous administration of symptom relieving drugs. Peripherally inserted central catheters (PICCs) and midline catheters (MCs) provide easy and accessible intravenous access. However, limited evidence supports the use of these devices in palliative care. The aim was to assess the use, safety, and efficacy of PICC and MC in this patient population.

**Methods:**

A retrospective study of all palliative care cancer patients who received PICC or MC at the Department of Palliative Medicine at Akershus University Hospital between 2020 and 2022.

**Results:**

A total of 374 patients were included; 239 patients received a PICC and 135 an MC with a total catheterization duration of 11,698 days. The catheters remained in place until death in 91% of patients, with a median catheter dwell time of 21 days for PICCs and 2 days for MCs. The complication rate was 3.3 per 1000 catheter days, with minor bleeding and accidental dislocation as the most common. The catheters were utilized primarily for opioids and other symptom directed treatments, and 89% of patients received a patient or nurse-controlled analgesia pump. Patients with PICC or MC discharged to home or nursing homes spent 81% of their time out of hospital.

**Conclusion:**

PICC and MC provide safe parenteral access for palliative care cancer patients where intravenous symptom treatment is indicated. Their use can facilitate intravenous symptom treatment beyond the confines of a hospital and supplement the traditional practice relying on subcutaneous administration.

## Introduction

In palliative care, the traditional route for parenteral drug administration is subcutaneous [1, 2]. However, the intravenous route has several potential advantages in this setting, including a faster onset of drug action, greater flexibility in injection volumes, drug and excipient use, less local infiltrations and irritation, and more predictable pharmacokinetics [3–5]. Peripheral venous access can be challenging to obtain and maintain in palliative care cancer patients. The use of peripherally inserted central venous catheters (PICC) and midline catheters (MC) allows for easy administration of intravenous medications and provision of regular care in both hospital and home settings, potentially reducing the burden on the patient and healthcare system compared to peripheral venous access [6, 7].

PICCs are inserted in the basilic, cephalic, or brachial vein without general anesthesia or a surgical incision, and their presence reduces the discomfort of frequent venipuncture and cannulation. The MC is a peripheral intravenous line measuring 3–8 inches and is inserted using a similar procedure, but with its tip not advanced beyond the distal axillary vein. The MC is indicated for short-term use (< 30 days). Removal of PICC or MC is a simple, safe procedure that can, if needed, be carried out at home [8].

There are several studies on PICC placement in cancer patients with the indication to maintain chemotherapy and/or nutrition therapy [9, 10]. These studies suggest PICCs are generally safe and convenient and can substitute other centrally or peripherally inserted venous devices. Based on systematic reviews and expert opinions, The Michigan Appropriateness Guide for Intravenous Catheters [8] states that a PICC could be indicated for palliative treatment during end-of-life care. Tripathi et al. [11] recently conducted a systematic review on the use of MC in a wide patient population. The review demonstrated complication rates for MC comparable to PICC but included only one study conducted in a palliative care unit [7]. In this study, the procedure was well tolerated, and the quality of care measured with the Palliative care Outcome Scale improved after catheter insertion, likely caused by improved symptom treatment.

Guidelines for symptom treatment in palliative care are based on the subcutaneous route [1, 2, 12] but advocate the intravenous route when subcutaneous administration is likely to fail, rapid pain control is needed or the patient already has a central venous catheter (CVC). The growing access to PICC and MC facilitates intravenous treatment in home care settings, making these methods increasingly popular in palliative care. However, there is limited evidence to support this practice. Therefore, this study is aimed at assessing the use, safety, and efficacy of PICC and MC in palliative care cancer patients admitted to a specialist palliative care hospital unit.

## Methods

### Study design

This is a retrospective observational study.

### Study population and setting

This study was conducted at the Department of Palliative Medicine, Akershus University Hospital (Ahus), which has a 12-bed palliative care ward, and serves a catchment area of 594.000. All adult cancer patients who had received a PICC or MC during admission at the Department of Palliative Medicine from January 2020 to December 2021 were included.

PICC and MC were inserted ultrasound guided by specially trained nurse anesthetists to ensure that best insertion practice was followed. The PICCs used were the 1 lm 4Fr PowerPiccSolo. The MCs used were 1 lm 4Fr Vygon and 1 lm 18G PowerGlide catheters.

### Data collection and management

Patients were identified from the medical records based on ICD-10 code for cancer (C00-C97) and intervention codes for insertion of a PICC or MC (PHX 17, PHX 25). Based on their first catheter insertion, the study population was stratified into two groups, as either PICC or MC. All subsequent CVC insertions were recorded for each patient, and each insertion was treated as an independent data point for outcome analysis. Patients who had a central venous line prior to admission were excluded due to expected insufficient documentation. Patients still alive at the time of data extraction in October 2022 were excluded.

We described patient demographics in accordance with the European Association for Palliative Care (EAPC) list of characteristics that describes palliative care patients (EAPC basic dataset) [13]. Present anticancer therapy was registered as ongoing if continuation of chemotherapy, immunotherapy, or radiotherapy was planned at time of PICC or MC placement. Only diagnoses and treatment documented in the patient’s medical records could be considered for registration.

Karnofsky performance status (KPS) [14] was registered at time of catheter insertion as either 50 or above or below 50. KPS is registered as less than 50 when a patient is unable to care for self and in need of institutional or equivalent care. When performance status was registered in the patient files, that value was recorded. In patients lacking KPS, a score was estimated based on the description of the patient's performance status.

Indications for PICC and MC placement, oral and parenteral medication before and after PICC/MC placement, data regarding the procedure, the total days of PICC/MC use, the type and time of catheter related complications, and the reasons for PICC removal were collected. Overall survival was measured from the time of PICC/MC placement to the time of death. Catheter complications were registered as described in the electronic records. Any suspected catheter-related infections were registered as a catheter related infection even if further diagnostics were not performed. Catheter occlusion, arm swelling, or pain was registered as a thrombosis/occlusion even if further diagnostics were not performed.

### Statistical analyses

Continuous variables were summarized as median and categorical variables were described using frequency distributions. The chi-squared test or Fisher’s exact test when appropriate was employed to assess the association between two categorical variables. The Wilcoxon-Mann–Whitney test (for two groups) was used to compare continuous variables. Survival was presented with Kaplan–Meier curves and compared using the log-rank test.

All analyses were conducted using IBM SPSS Statistics 28.0.1.1 software.

### Research ethics

A Remit Assessment Form was submitted to the Regional Ethics Committee, which approved the project as a quality assurance project. The study was approved by the Data Protection Officer at Ahus.

## Results

### Study population

From January 2020 to December 2021, 948 individual patients were admitted to the Department of Palliative Medicine. A PICC or MC intervention code was present in 401 individual medical records. Among these 374 individual patients were included in the study with a total catheterization duration of 11,698 days. Patients incorrectly registered as admitted to the department (*n* = 5), patients with a CVC in place prior to admission (*n* = 10), and patients alive at time of data extraction (*n* = 12) were excluded.

The median (range) age of patients was 70 (28–97) years, and 50% were female (Table 1). The majority of patients (93%) had metastatic disease. The most common cancer diagnoses were malignant neoplasms of digestive organs (35%) and respiratory organs (21%). Most patients (74%) had stopped all anticancer therapy at time of catheter insertion, and 74% of the patients had a Karnofsky status below 50.

**Table 1:** Baseline characteristic

Characteristics		Total *N* = 374		PICC *N* = 239		MC *N* = 135		*p*-value
		*N*	(%)	*N*	(%)	*N*	(%)	
Age, median (range) (IQR)		70	(28–97) (60–79)	69	(28–97) (59–75)	72	(39–95) (62–77)	0.024
Gender	Female	186	(50)	109	(46)	77	(57)	0.034
Performance status	Karnofsky < 50	269	(74)	151	(63)	118	(87)	< 0.001
Metastatic situation								
Localized/locally advanced disease only		27	(7.2)	16	(6.7)	11	(8.1)	
Metastatic disease		347	(85)	223	(93)	124	(92)	
Cancer diagnose								
Digestive organs		131	(35)	77	(32)	54	(40)	
Respiratory and intrathoracic organs		79	(21)	50	(21)	29	(22)	
Urinary tract		29	(7.8)	22	(9.2)	7	(5.2)	
Male genital organs		28	(7.5)	22	(9.2)	6	(4.4)	
Breast		25	(6.7)	15	(6.3)	10	(7.4)	
Female genital organs		18	(4.8)	13	(5.4)	5	(3.7)	
Ill-defined, secondary, and unspecified sites		16	(4.3)	10	(4.2)	6	(4.4)	
Melanoma and other malignant neoplasms of skin		15	(4.0)	10	(4.2)	5	(3.7)	
Other		33	(24)	20	(8.4)	13	(9.6)	
Any ongoing anticancer treatment		70	(22)	42	(18)	28	(20.7)	0.027
Chemotherapy		42	(11)	35	(15)	7	(5.2)	
Immunotherapy		12	(3.2)	13	(3.8)	4	(3.0)	
Hormone therapy		17	(4.5)	9	(3.8)	3	(2.2)	
Radiotherapy		9	(2.4)	9	(2.1)	8	(5.9)	
PICC/MC insertion vein								
Basilic vein		286	(77)	194	(81)	92	(68)	
Brachial vein		65	(17)	40	(17)	25	(19)	
Cephalic vein		15	(4.0)	3	(1.3)	12	(8.9)	
Not stated		8	(2.1)	2	(0.8)	6	(4.4)	
PICC/MC insertion side	Right arm	322	(87)	217	(91)	105	(78)	< 0.001

*PICC* peripherally inserted central catheter, *MC* midline catheter, *IQR* interquartile range

Of the 374 patients in the study, 239 (64%) patients received a PICC and 135 (36%) patients received an MC. Patients who received an MC had a lower performance status (*p* < 0.001) and less ongoing anticancer treatment (*p* < 0.001) than patients who received a PICC. Catheters were most commonly placed in the right arm (87%) through the basilic (76%) or brachial vein (17%).

### Utilization

Referral indications and actual catheter utilization for the 374 patients are shown in Table 2. Regular or as needed intravenous medication for symptom treatment was a referral reason in 98% of patients. The need for an intravenous patient or nurse controlled analgesia/infusion pump (PCA-pump) was an indication in 76% of referrals. The option to do blood sampling from PICC or MC was an added indication in 64% of patients. Hydration or parenteral nutrition was a referral reason for 5.5% of patients, and tumor-targeted treatment was one of the indications in 1.1% of patients.

**Table 2:** Indications given for catheter placement and actual utilization

Catheter utilization *N* = 374*	Indications		Actual utilization	
	*N*	(%)	*N*	(%)
IV regular/as needed medication for symptom treatment	367	(98)	352	(94)
PCA-pump	285	(76)	331	(89)
Blood sampling	240	(64)	158	(42)
Hydration/nutrition	19	(5.5)	33	(9.5)
Tumor targeted treatment	4	(1.1)	4	(1.1)
No registered utilization			4	(1.1)

*IV* intravenous, *PCA* patient or nurse controlled analgesia/infusion pump

*Potentially more than one indication for each patient

Before catheter placement, 53% of patients already had one or more PCA-pumps. Following catheter placement, 331 (89%) of patients received PCA-pumps. Of these patients, 48% periodically utilized more than one PCA pump.

Following catheter placement, intravenous opioids were administered to 91% of the 374 patients, most commonly through a PCA-pump (86%) (Table 3). Intravenous sedatives were administered with a PCA-pump in 45% of patients, and another 21% received sedatives as intravenous injections. Intravenous antibiotics including treatment of oral candidiasis was provided for 19% of patients. Between 13 and 19% of patients received intravenous antacids, corticosteroids, and antiemetics. Palliative sedation with propofol infusion was provided for 12 patients (3.2%). After catheter placement, 22% of patients did not receive any oral medication.

**Table 3:** Medication administered IV after PICC or MC placement

Medication (*N* = 374)*	IV		IV PCA	
	*N*	(%)	*N*	(%)
Opioids	17	(4.5)	321	(86)
Sedatives/anxiolytics	80	(21)	169	(45)
Drug(s) for acid related disorders	71	(19)	1	(0.3)
Antibiotics	71	(19)		
Corticosteroids	51	(14)		
Antiemetics	50	(13)		
Non-opioid analgesics	16	(4.3)		
Neuroleptics	5	(1.3)	8	(2.1)
Propofol continuous infusion			12	(3.2)
Diuretics	10	(2.7)		
Other	20	(5.3)	15	(4.0)

Empty cells indicate *N* = 0

*IV* intravenous, *PCA* patient or nurse controlled analgesia/infusion pump, *PICC* peripherally inserted central catheter, *MC* midline catheter

*Potentially more than one medication for each patient

### Catheter dwell time and complications

**Fig. 1:**
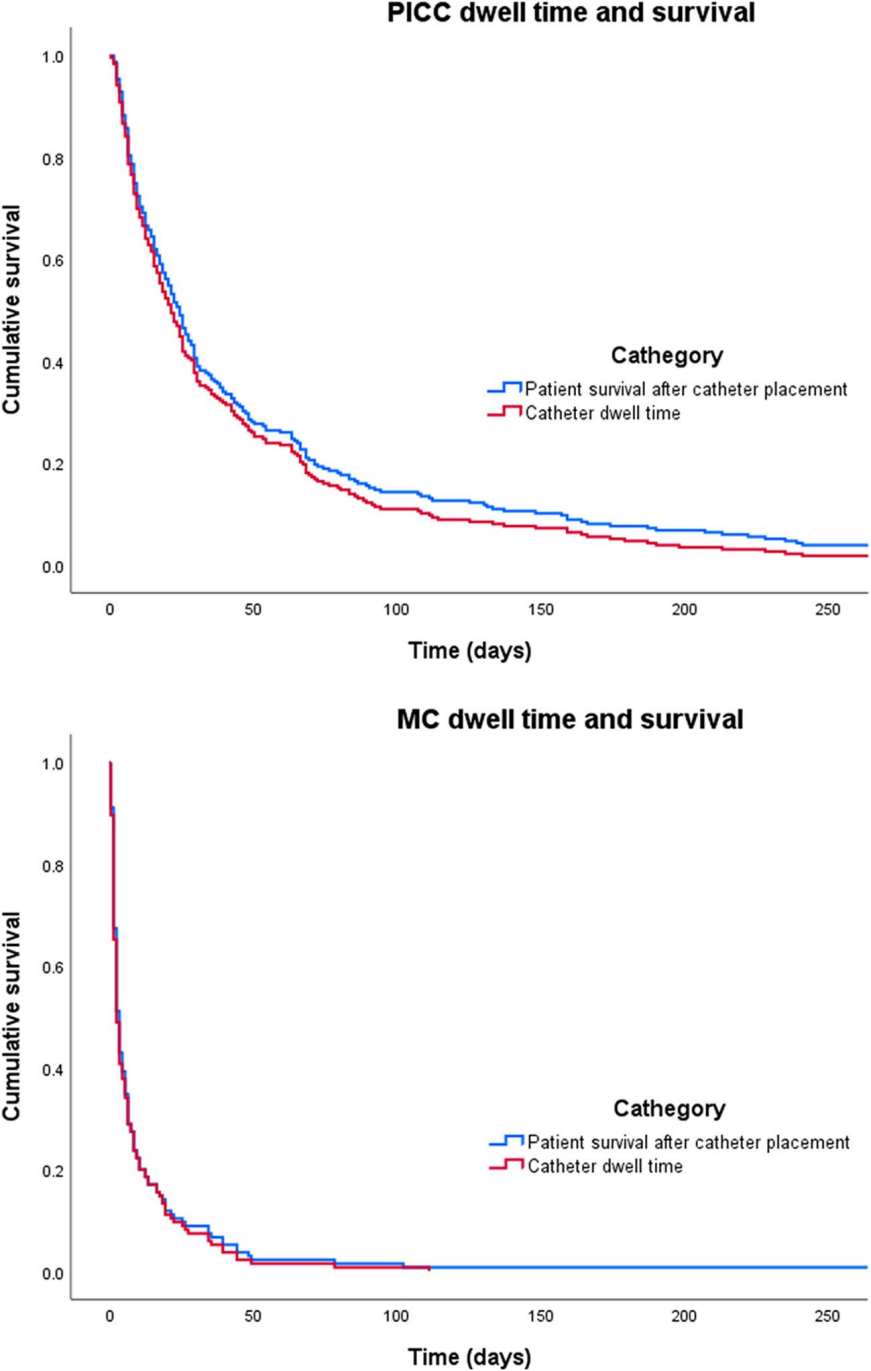
Patient survival and catheter dwell time for PICC and MC

Patient survival and catheter dwell time are shown in Fig. 1. The first established catheters were kept until time of death for 339 patients (91%). The median (IQR) survival after PICC placement was 24 (8–63) days and after MC 3 (1–8) days (*p* < 0.001). The catheter dwell time for PICC and MC was, respectively, 21 (8–53) and 2 (1–8) days (p < 0.001).

In eight patients, the catheters were removed due to no further need. Ten patients with MC kept their catheter for more than the recommended 30 days. No complications were observed in these patients. In the PICC group, eleven patients kept their catheter for more than six months.

The overall complication rate was 3.3 per 1000 catheter days. The rate was higher for the MC group (7.2) compared to PICC (2.9) (*p* = 0.038) (Table 4). Complications overall occurred at median (IQR) 13 (1–50) days post PICC and 2 (0–19) days post-MC placement.

**Table 4:** Complications after PICC or MC placement

	Total *N* = 374		PICC *N* = 239		MC *N* = 135	
Complications	*N*	(%)	*N*	(%)	*N*	(%)
Any complications						
Accidental dislocation	13	(3.5)	10	(4.2)	3	(2.2)
Hemorrhage	9	(2.4)	7	(2.9)	2	(1.5)
Occlusion/thromboses	7	(1.9)	6	(2.5)	1	(0.7)
Infection	4	(1.1)	4	(1.7)	0	(0.0)
Irritation	3	(0.8)	3	(1.3)	0	(0.0)
Leakage	3	(0.8)	1	(0.4)	2	(1.5)
Total	39	(11)	31	(13)	8	(5.9)
Complication leading to removal						
Accidental dislocation	12	(3.2)	9	(3.8)	3	(2.2)
Hemorrhage	1	(0.3)	1	(0.4)	0	(0.0)
Occlusion/thromboses	5	(1.3)	4	(1.7)	1	(0.7)
Infection	4	(1.1)	4	(1.7)	0	(0.0)
Irritation	3	(0.8)	3	(1.3)	0	(0.0)
Leakage	3	(0.8)	1	(0.4)	2	(1.5)
Total	28	(7.5)	22	(9.2)	6	(4.4)
Total catheter days	11698		10583		1115	
Complication per 1000 catheter days (95% CI)	3.3	(2.3–4.4)	2.9	(1.9–4.0)	7.2	(6.1–10.6)
Time to complication median (IQR)	11	(1–27)	13	(1–50)	2	(0–19)

**Fig. 2:**
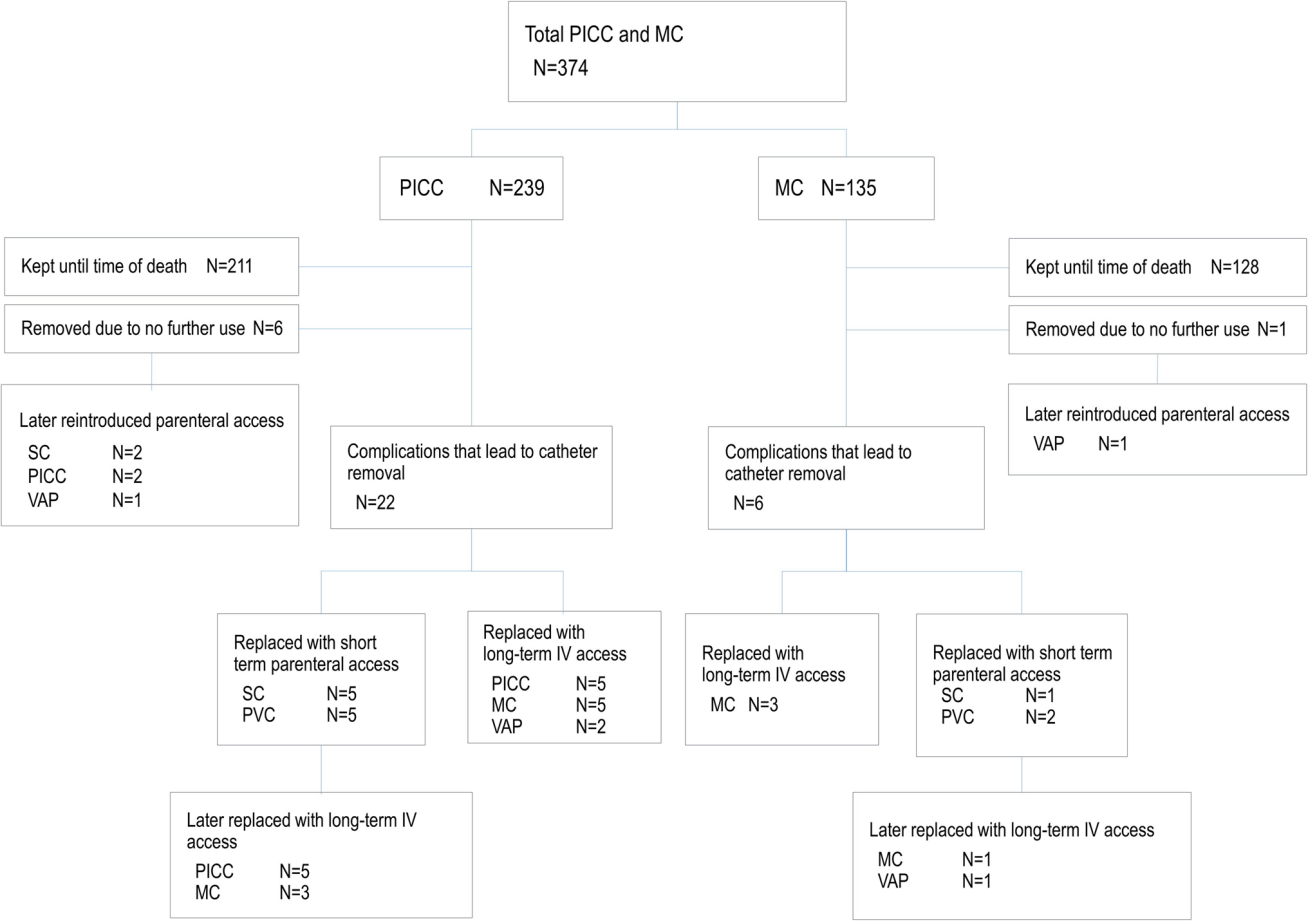
Catheter removal and renewal chart

Catheter = related complications were reported in 39 patients and resulted in catheter removal for 28 of these patients (Table 4). After catheter removal, 25 of these patients received a new PICC, MC, or VAP directly (15 patients) or after a period of subcutaneous or short term intravenous access (10 patients) (Fig. 2).

One of the patients with a second PICC needed a third and fourth catheter due to occlusion and later infection. One patient with PICC died 71 days after catheter placement with symptoms of a catheter related sepsis confirmed by positive blood and catheter tip culture of *Staphylococcus aureus*. Intensive care unit admission was discussed but not initiated due to terminal stage cancer.

Hemorrhage and accidental dislocation occurred the same day as catheter placement in eight patients. Hemorrhage was controlled with dressings and compression without removing the catheter in all but one patient.

### Hospitalization after catheter placement

Of patients who received an MC, 68% were hospitalized until death (Table 5). Patients who received a PICC were more often discharged to home (49%, *p* < 0.001). Patients with a PICC or MC eventually died in hospital in 57% of cases. Patients with an MC more often died in hospital than patients with a PICC (*p* < 0.001). Of the 233 patients who were discharged alive after catheter placement, 51% had no readmissions to hospital. Patients discharged alive after PICC or MC placement spent median (IQR) 81% (70%–91%) of remaining lifetime outside the hospital, i.e., at home or in nursing home. Regular injections that had been established during admission were consistently maintained in home care or nursing homes, and 81% of patients discharged alive had a PCA-pump.

**Table 5:** Discharge pattern after PICC or MC placement

Discharge pattern		Total *N* = 374		PICC *N* = 239		MC *N* = 135		*p*-value
		*N*	(%)	*N*	(%)	*N*	(%)	
Discharged	As dead	141	(38)	49	(21)	92	(68)	< 0.001
	Home	130	(35)	117	(49)	13	(10)	< 0.001
	Nursing home/hospice	96	(26)	69	(29)	27	(20)	
	Other hospital	7	(1.9)	4	(1.7)	3	(2.2)	
Died in hospital at current or later admission		214	(57)	114	(48)	100	(74)	< 0.001

*PICC* peripherally inserted central catheter, *MC* midline catheter

## Discussion

The PICC and MC catheters were safely inserted and had few complications in palliative care cancer patients, with accidental catheter dislocation and minor bleeding as the most common complications. The patients had advanced disease, low performance status, and short life expectancy reflected in the short catheter dwell time and patient survival after PICC and especially MC placement. Importantly the catheters were in place until death in 91% of patients, indicating that PICC and MC is a feasible solution for parenteral drug administration in palliative care patients with advanced disease.

The present finding of low complication rates aligns with previous studies on palliative care cancer patients earlier in their disease trajectory. [9–11]. Former studies have primarily focused on providing intravenous access for life prolonging treatment such as tumor targeted treatment, antibiotics, hydration, and nutrition. These studies have demonstrated that PICC and MC were well tolerated and preferred by patients over repeated peripheral vein punctures, with complications occurring in a minority of cases. The present study suggests that PICC and MC could provide safe and effective intravenous access also for late-stage cancer patients requiring parenteral administration of symptom relieving drugs.

The catheters were used in line with referral reasons, primarily for analgesia and sedatives with a PCA-pump. Intravenous nutrition and hydration were infrequent in our study population indicating that a venous access is not seen as an opportunity to intensify life prolonging and perhaps futile treatment in end-stage palliative care.

Catheter dwell time and patient survival after catheter placement have a large range in the literature. Catheters placed early in the disease trajectory primarily for tumor-targeted treatment are typically kept for several months [10]. Studies on terminally ill cancer patients [15–17] report a PICC dwell time in the same range as our study.

The complication rate for PICC catheters in this study (2.9 per 1000 catheter days) was higher than the rate reported in a large retrospective study of PICC catheters in cancer patients receiving chemotherapy [10] (1.09 per 1000 catheter days). However, that study did not include accidental catheter removal as a complication, which may account for the difference in findings. The short dwell time for MC in our study, contributes to a low number of complications but a relatively high complication rate per 1000 catheter days. The proximity of MC insertion to the time of death in the present study reflects a tradition at the Department of Palliative Medicine of requesting MC catheters to avoid repeated venipuncture and secure intravenous access during end of life care.

Estimation of life expectancy is challenging. Even experienced palliative care clinicians tend to overestimate [18]. This could influence the choice of an MC or PICC catheter: The low median dwell time for PICC where 62% of patients kept the catheter for a month or less, indicates that for many of these patients an MC would be adequate. Furthermore, the low median survival after MC placement suggests that a peripheral venous catheter could have been sufficient for some of these patients. This raises concerns about the potential for overtreatment due to the availability of interventions. There were no registered complications for patients with an indwelling MC for longer than the recommended duration of one month. This suggest that patients with short life expectancy and well-functioning MC may not need a renewed PICC or MC, even if the patient lives longer than expected. This further highlights the importance of prudent use of medical interventions. It is crucial to ascertain that treatments are employed judiciously even when they are easily available.

Subcutaneous administration is traditionally accepted as a good enough parenteral route for symptom relief in palliative care cancer patients. Our research findings may reflect a shift in clinical practice towards the intravenous route. The clinicians’ emerging preference for the intravenous route for pain and symptom management in palliative care should be explored further to see if this relates to better symptom treatment or simply represents an unnecessary complication of current practice guidelines. Despite receiving intravenous infusions, patients in our study spent most of their remaining lifetime at home or nursing homes demonstrating that intravenous treatment no longer is limited to hospitalized patients.

A major strength of this study is the inclusion of a complete cohort of patients receiving PICC and MC catheter at a large department of palliative medicine that serves a catchment area of one-tenth of the Norwegian population. Complete hospital records and charts were thoroughly reviewed adding to a high internal validity.

One important limitation in the present study is the single-center study design where hospital traditions including the institutional culture for intravenous symptom relief with MC or PICC in end of life care could affect external validity. Potentially incomplete records and missing data limit the retrospective study design based on medical records. Even though IV PCA-pumps and other established infusions were routinely continued after discharge, there was no direct access to records from nursing home or home care, therefore events occurring after discharge that were not reported to the Department of Palliative Medicine might have been missed. The low median dwell time for MC in this material prohibits drawing solid conclusions regarding the complication profile. Patients in their final days of life are spared examinations and diagnostics not resulting in any meaningful therapy. Consequently, complications like thrombosis and infection might have been missed, even though medical history or findings that indicated such complications, also without diagnostic verification, were counted as a complication in our study.

Despite these limitations, the present findings indicate that PICC and MC are safe and efficient parenteral access devices for palliative care cancer patients where continuity of intravenous symptom treatment is seen as an advantage, and can provide a robust option for intravenous symptom treatment outside the hospital setting where practice traditionally has relied on subcutaneous infusions.

## Data Availability

The data that support the findings of this study are not openly available due to reasons of sensitivity and are available from the corresponding author upon reasonable request. Data are located in controlled access data storage at Akershus University Hospital.
